# Lower-Extremity Arteriovenous Malformation Masquerading as Peripheral Artery Disease

**DOI:** 10.7759/cureus.41431

**Published:** 2023-07-05

**Authors:** Briana Sylvester, Niteesh Sundaram, Silviu Marica, Burt Cagir, Joseph Ronsivalle

**Affiliations:** 1 School of Medicine, Geisinger Commonwealth School of Medicine, Scranton, USA; 2 General Surgery, Crozer-Chester Medical Center, Upland, USA; 3 Vascular Surgery, Guthrie Clinic/Guthrie Robert Packer Hospital, Sayre, USA; 4 Colorectal Surgery, Guthrie Robert Packer Hospital, Sayre, USA; 5 Interventional Radiology, Guthrie Robert Packer Hospital, Sayre, USA

**Keywords:** transarterial embolization, arteriovenous malformation, peripheral arterial disease, interventional radiology, vascular surgery

## Abstract

An arteriovenous malformation (AVM) refers to an anomalous, direct connection between an artery and a vein. Typically, these two vessels are interposed by high-resistance capillary beds, the absence of which results in a high-flow system from an artery into a vein. Venous vessel walls are not designed to handle such high-pressure blood flow, and their vessel wall structure becomes permanently altered and weakened. For this reason, AVMs are at an increased risk for hemorrhages and ruptures. AVMs present with a spectrum of symptoms, ranging from skin discoloration, ischemia in distal tissues, and heart failure from arteriovenous shunting. Here, we present a case of a patient who underwent amputation of his second left toe, initially thought to be a result of peripheral arterial disease (PAD) due to his extensive smoking history. Further imaging findings revealed a rare lower-extremity AVM as the source of his ischemia, not PAD. Ultimately, the treatment of the vascular anomaly and resolution of the patient’s symptoms were achieved via transarterial embolization. This case emphasizes the importance of looking beyond PAD as the cause of distal lower-extremity ischemia and provides insights into an uncommon and often missed diagnosis of extracranial AVMs.

## Introduction

An arteriovenous malformation (AVM) is a rare, aberrant vascular connection between an artery and a vein that bypasses a capillary system. AVMs are at risk of hemorrhage from inherent vulnerabilities in their altered vessel wall structure and due to damage of vessel architecture from high-pressure arterial flow against low-pressure venous wall structures [1,2]. The percentage of previously unruptured AVMs that result in hemorrhage annually is approximately 2%, which increases to over 4% in the case of recurrent hemorrhage [3]. AVMs present with a spectrum of symptoms, ranging from bluish discoloration of the skin, ischemia in distal tissues, pain, weakness, and heart failure from arteriovenous shunting, as the AVM continues to grow in size [4]. Here, we present a case of a patient who underwent amputation of his second left toe, initially thought to be a result of peripheral arterial disease (PAD). A diligent workup of this patient’s chief complaint revealed that his arterial insufficiency resulted from an AVM, not PAD.

This article was previously submitted as a meeting abstract to the 6th Annual Guthrie Research Day on April 28, 2023.

## Case presentation

A 43-year-old male, whose only past medical history included a vasectomy and 20-pack-year smoking history, presented to our vascular surgery clinic. He reported a five-month history of left second toe pain associated with numbness, tingling, and a burning sensation. Before being seen in our office, he sought treatment at an outside facility, resulting in a diagnosis of mild PAD and resection of an ingrown toenail. Unfortunately, the patient had continued pain and developed dry gangrene from non-healing wounds at the level of his resected toenail as a result of microtrauma from the previous toenail resection.

Evaluation at our vascular clinic included pulse volume recordings (PVRs), ankle brachial index (ABI) and toe brachial index (TBI), computed tomography angiography (CTA) of the abdomen with runoff to evaluate for sources of atherosclerotic embolization, and magnetic resonance imaging (MRI) of the left foot to evaluate for osteomyelitis in the setting of gangrene.

**Figure 1:**
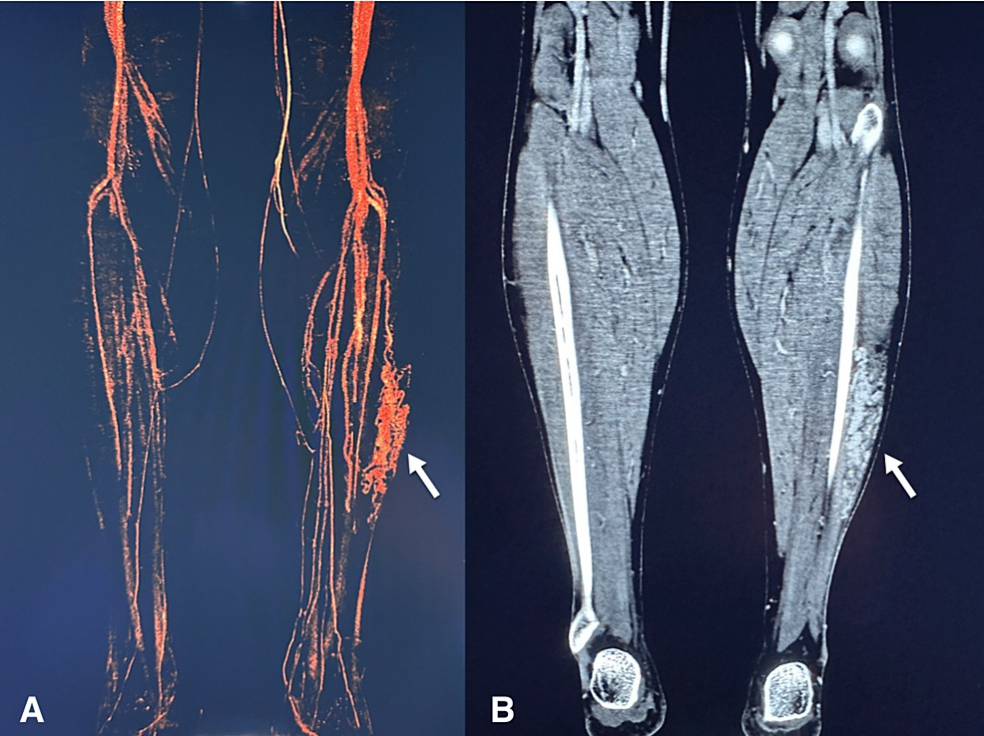
CTA maximum intensity projection rendering of the lower-extremity vasculature shows an intramuscular arteriovenous malformation (arrows) of the lateral left calf with evidence of an early venous return. CTA, computed tomography angiography

The patient’s PVRs showed normal waveforms to the ankle and mild waveforms of the left first and second toes. The left posterior tibial artery Doppler was biphasic, and the left dorsalis pedis artery Doppler was biphasic to triphasic. Bilateral ABIs were within normal limits (right ABI: 1.14; left ABI: 1.03). By contrast, the left toe brachial index was suggestive of mild distal PAD (left TBI: 0.69; right TBI: 0.90). The MRI results did not suggest ongoing osteomyelitis. However, the lack of soft tissues overlying the distal phalanx of the affected toe necessitated amputation, which was performed at our institution. Final pathology from the amputation revealed fragments of slightly tangentially sectioned hyperparakeratotic skin with evidence of ischemic necrosis and secondary ulceration, decalcified bone and bone at the resection margin appearing negative for acute osteomyelitis, portions of the skin at the resection margin appearing viable, and separate fragments of dense tendon-like fibroconnective tissues with no specific histopathologic abnormality. The CTA of the patient’s abdomen, pelvis, and lower extremities showed a normal abdominal aorta and patent iliac, femoral, and popliteal arteries. However, the CTA also revealed a vascular malformation overlying the midshaft of the fibula and within the left fibularis brevis and longus muscles, measuring 3.4 cm x 2.7 cm x 10.8 cm (Figures 1A, 1B). The patient was referred to interventional radiology (IR) for the treatment of his symptomatic AVM.

**Figure 2:**
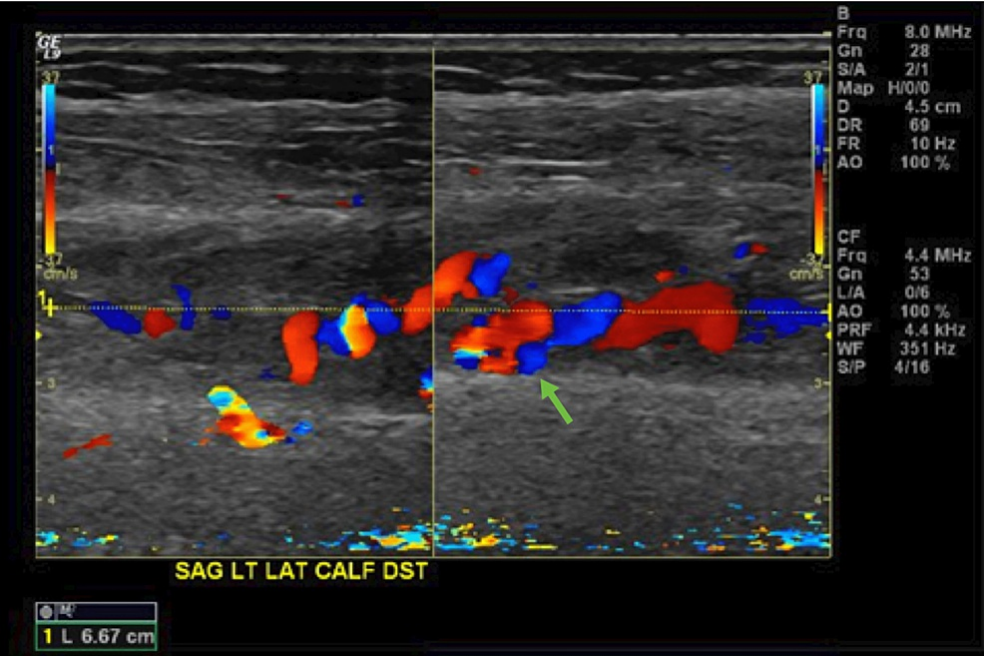
Duplex ultrasound of the left lower-extremity arteriovenous malformation demonstrating an abnormal intramuscular vasculature (arrow).

**Figure 3:**
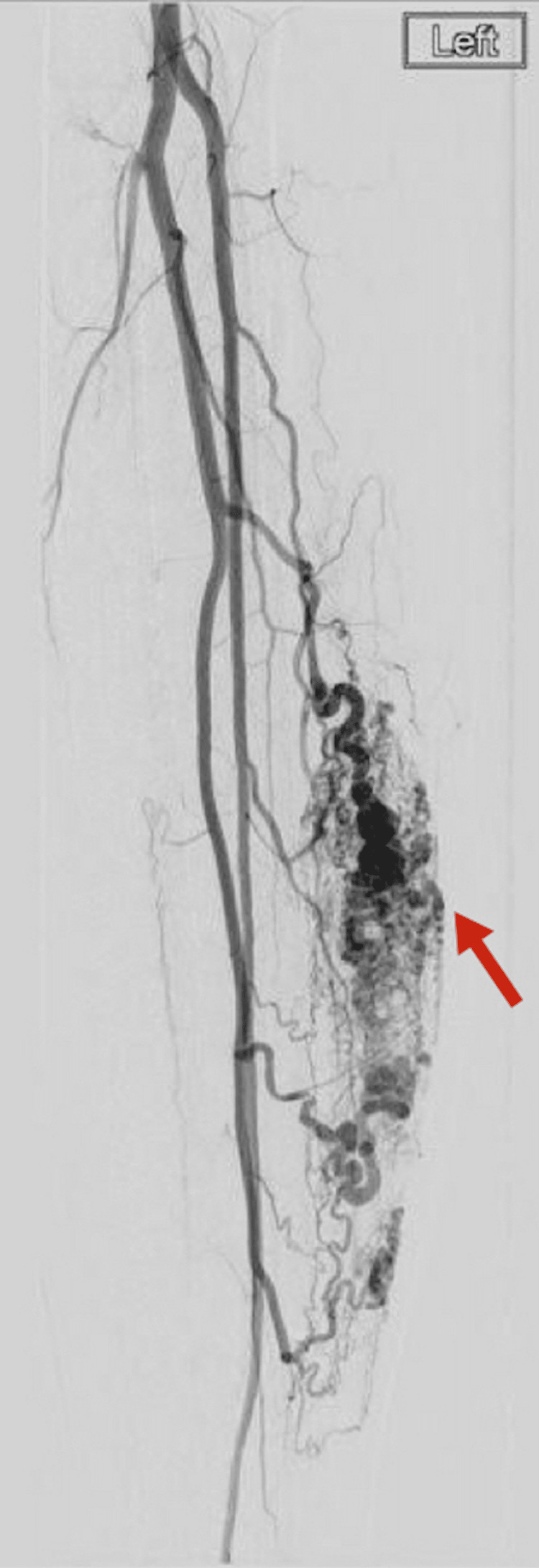
Pre-embolization angiogram of the left lower-extremity arteriovenous malformation demonstrating multiple feeding vessels (arrow).

Duplex imaging was ordered to further characterize the vascular malformation's flow quality. Ultrasound revealed triphasic flow throughout the lower left extremity and low-resistance arterial flow within the AVM (Figure 2). The patient’s persistent pain, edema, and erythema post-amputation were thought to be the result of venous reflux and congestion. The patient underwent transarterial embolization with ethylene-vinyl alcohol copolymer (Onyx Liquid Embolization System) and embolization coils via a microcatheter. Angiography findings included a large, complex left lower-extremity AVM with most of its feeding vessels derived from the anterior tibial, peroneal, and posterior tibial arteries (Figure 3). Embolization and polymerizing agent placement were directed at these arterial contributors: two feeding branches from the anterior tibial artery, the terminal vessel of the peroneal artery, and a distal feeding branch of the posterior tibial artery.

**Figure 4:**
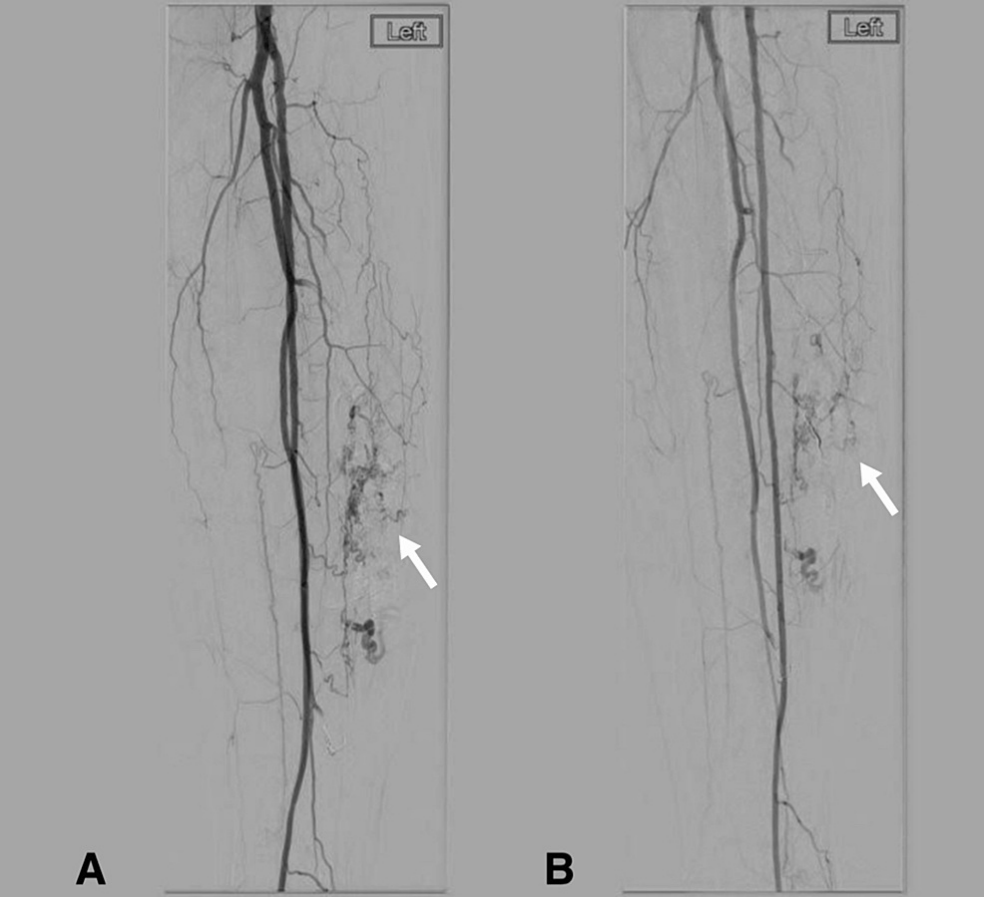
Post-embolization angiogram of the left lower-extremity arteriovenous malformation demonstrating a significantly decreased vascular supply (arrows).

A final angiogram showed significantly reduced flow to the AVM (Figures 4A, 4B). There were feeders to the AVM from smaller vessels and drainage from two distinctly visible veins. However, this was considered an adequate embolization for treatment. The patient tolerated the procedure well, without complication, and was discharged the same day with plans for a one-week telephone follow-up and a two-month office follow-up with ultrasound. Unfortunately, the patient was lost to follow-up.

## Discussion

AVMs are progressive, congenital vascular anomalies that expand into adolescence and adulthood. Although the exact cause of AVMs is unknown, they can occur sporadically or as a part of a syndrome, such as Cobb’s syndrome or hereditary hemorrhagic telangiectasia (Osler-Weber-Rendu syndrome) [1]. Though typically intracranial, common sites of extracranial AVMs include the head, neck, trunk, and extremities [3,4]. The true prevalence of AVMs remains unknown. However, there are approximately 0.89 to 1.34 cases per 100,000 person-years [5]. The expansion of AVMs can cause bleeding, disfigurement, neurovascular compromise, pain, and, rarely, cardiac insufficiency [3,4,6].

Physical examination may reveal edema, hyperhidrosis, audible bruit, palpable thrill, or tissue change resulting from arteriovenous shunting. In severe cases, signs of cardiopulmonary overload may be present [4,7]. While digital subtraction angiography is considered the gold standard of AVM diagnosis, magnetic resonance angiography (MRA) and CTA often suffice, showing characteristic flow-associated signal voids or shunts into vessels surrounding the nidus of the AVM [3,4,7]. The flow velocity and AVM extent are characterized using Doppler ultrasound and the degree of expansion with the Schobinger scale (Table 1) [8]. Key differential diagnoses include hamartoma, hemangioma, lymphatic malformation, venous malformation, and capillary malformation [3]. Given their progressive nature and tendency for recurrence, treatment of AVMs often requires a multidisciplinary approach [1,2].

**Table 1:** Schobinger clinical classification of arteriovenous malformations.

Stage	Characteristics
Quiescence (I)	Largely asymptomatic; can have warmth of affected tissues; detectable on Doppler
Expansion (II)	Enlargement of lesion; audible bruit; palpable thrill; tortuous veins
Destruction (III)	Ischemic changes; ulcers; tissue necrosis; poor wound healing; lytic bone lesions
Decompensation (IV)	High-output heart failure; venous hypertension

Treatments include conservative management, pharmacologic trials, transarterial embolization, stereotactic gamma knife radiosurgery, and open surgical resection of the affected tissue [1,7]. Depending on the size, site, and extent of the lesion, the goal of treatments may be the temporary inhibition of AVM progression rather than cure [4,9-11]. Based on the Schobinger scale, stage I AVMs rarely require interventions and can be managed conservatively, stage II AVMs can be considered for invasive management, and stage III/IV AVMs require either embolization or surgical management [8]. Conservative management includes serial examinations and imaging or compressive dressings for symptom management and is appropriate for asymptomatic or mildly symptomatic lesions [7]. The role of pharmacotherapy in the management of AVMs remains controversial. Targets of vascular endothelial growth factors (VEGFs), such as bevacizumab, have demonstrated limited response and, in some cases, the development of therapeutic resistance [7].

For symptomatic AVMs, invasive management in the form of endovascular embolization or operative management is the mainstay of treatment. Unfortunately, even with invasive treatment, AVMs can reoccur, with Schobinger stage III/IV AVMs being more likely to reoccur after treatment than Schobinger stage I/II AVMs [7,8]. Surgical management has shown to be very effective for smaller, well-localized lesions, but it is a poor treatment modality for diffuse and more extensive lesions [7].

In addition, surgical management is associated with higher complication rates, such as uncontrolled bleeding and increased morbidity [12]. Transarterial embolization provides a minimally invasive approach with low complication rates that may benefit patients by shrinking the size of AVMs preoperatively and can achieve adequate treatment for many with localized AVMs [4,13-15]. A multidisciplinary approach to embolization and surgery can improve operative outcomes and reduce recurrence rates [12]. The target of surgical resection and embolization is occlusion and/or resection of the low-resistance nidus of the AVM, at which supplying branches converge [4,7].

Our patient presented to the clinic with a smoking history and chronic non-healing wound, dry gangrene, and pain suggestive of PAD. On physical exam, there was no evidence of superficial AVM features or size discrepancies between both calves. Multimodal imaging studies elucidated an AVM as the etiology of his chronic toe pain. Given that our patient’s non-invasive testing did not indicate that an occlusive vessel disease was the cause of his distal ischemia, we hypothesize two possible mechanisms that resulted in his toe gangrene. The first mechanism is arterial steal due to the presence of an AVM. The AVM provides a low resistance pathway for blood to flow, which results in blood being diverted from the distal foot. This explains why our patient was experiencing numbness and pain in his distal second toe. As to why only the second toe was affected could be related to the amount of blood flow each toe is receiving on a more microvascular level than can be appreciated on imaging or non-invasive studies [16]. The second mechanism is a possible thromboembolic event. There have been reported cases of AVMs spontaneously thrombosing given the tortuosity of the vasculature serving as a nidus for clot formation [17]. Furthermore, AVMs allow for a direct connection between the arterial and venous system, which means that a clot from a calf vein could have broken off and entered the arterial system directly via the patient’s AVM [18]. Our workup of the patient did not reveal any evidence of venous clots in his lower extremity or thrombosed segments of his AVM, but this could have occurred prior to the patient’s presentation in our clinic and resolved by the time we evaluated him.

Our intervention radiology colleagues were able to intervene effectively in the case of this patient’s symptomatic AVM. This case highlights the significance of a comprehensive investigation into patient complaints despite risk factors that would suggest a common diagnosis. The patient’s imaging findings were both an opportunity for documenting an uncommon occurrence of lower-extremity AVM and a means for a broader understanding of this patient’s chief complaint. Unfortunately, as this patient was lost to follow-up, we cannot definitively report his long-term outcome. The literature shows that although many peripheral AVMs can be treated successfully, the most common complications include bleeding, damage to nearby arteries and veins leading to distal ischemia and further tissue loss, and nerve injury leading to persistent pain [19].

## Conclusions

Our case highlights an atypical presentation of lower-extremity AVMs in a patient who presented with distal lower-extremity ischemia and whose risk factors initially suggested the common diagnosis of PAD. This case emphasizes the need for a diligent evaluation of all patients and provides insights into an uncommon and often missed diagnosis of extracranial AVM. Further clinical research is warranted to better understand AVMs' epidemiology and pathophysiology, their surgical management, and the long-term clinical outcomes of the affected patient population.
